# Subcategorisation of Data for AI Models in Healthcare: A Case Study in Mammography

**DOI:** 10.3390/diagnostics16152367

**Published:** 2026-07-28

**Authors:** Jessica E. Goldring, Elizabeth A. Cooke, Ruben van Engen, Alistair Mackenzie, Jenny Venton, Carlijn Roozemond, Spencer A. Thomas, Nadia A. S. Smith

**Affiliations:** 1 National Physical Laboratory, Teddington, Middlesex TW11 0LW, UK; 2 UCL Great Ormond Street Institute of Child Health, University College London, 30 Guilford St, London WC1N 1EH, UK; 3 Dutch Reference Centre for Screening (LRCB), Wijchenseweg 101, 6538 SW Nijmegen, The Netherlands; 4 Royal Surrey NHS Foundation Trust, Guildford GU2 7XX, UK; 5 TÜV SÜD UK, Chadwick House Warrington Rd, Birchwood Park, Risley, Warrington WA3 6AE, UK

**Keywords:** artificial intelligence, categorisation, training, mammography, metadata

## Abstract

**Background/Objectives:** Accurate data subcategorising is vital for reliability and traceability in the training and validation of all artificial intelligence (AI) models. **Methods:** In this paper we show the complexity of clinical and technical features likely to affect the appearance and interpretation of mammography images and in turn affect the output of AI software used to aid clinical decisions. **Results:** Using mammography as a case study, the equitability covers screened population characteristics (e.g., women’s age and ethnicity) and image acquisition key factors (e.g., brand of system, exposure factors, image processing). We examine some studies and available datasets of mammography images, summarising the metadata available. **Conclusions:** We recommend that, where possible, AI models are trained and evaluated using data that includes subcategories based on these features, ensuring increased equitability in the data and coverage of image heterogeneities; or, where not possible, that the subcategories for which the AI model is valid are clearly defined. Such practices can easily be implemented in a wide range of AI applications but are illustrated here with mammography. Clinical Relevance: Reliable AI holds invaluable potential for both clinical efficiency and accuracy in diagnosis. With appropriately categorised training data, a reduction in subjective assessment can be achieved, leading to trustworthy and rapid assessment.

## 1. Introduction

In the year 2022, 2.3 million women worldwide were diagnosed with breast cancer causing 670,000 deaths [1]. Due to limitations of the screening method, breast cancer can go undetected due to (i) low sensitivity of mammography in some subgroups, like women with dense breasts; (ii) cancer growth patterns resulting in subtle mammographic presentation or cancers with fast growth rates that outpace screening intervals and are therefore ‘missed’ at screening; and (iii) radiologists’ reading or interpretation errors. Artificial intelligence (AI) has great potential to address these challenges [2]. However, its use in breast cancer screening and indeed all healthcare [3] is subject to issues such as trustworthiness, robustness, generalisability, and interpretability. Accuracy and robustness are critical for all AI usage within a clinical setting; however, issues such as bias and poorly constructed training data [4,5] are significant barriers to clinical adoption. Another related problem is that of overfitting, with solutions ranging from naively adding more data, to the inclusion of regularisation terms in model training such as weight decay [6], weight dropout [7] and online data augmentations [8,9,10]. These methods can mitigate overfitting to a degree, but the lack of generalisability to unseen data is not solely due to model training schemes, but also poor categorisation of the training data where data instances may be inappropriately combined or excluded. This point is pertinent for healthcare data where instances have complex provenance and differences between the characteristics of the data, i.e., different categorisations, can differentiate between data points, e.g., disease progression of risk for age groups [10,11,12,13,14] and gender [15,16,17,18]. The FAIR (Findability, Accessibility, Interoperability, and Reusability) principles [19] are widely being adopted to tackle reproducibility and data visibility; however, these do not ensure the data are correctly or sufficiently categorised. Data curation with rich metadata is essential for reproducible science [20] but also confident data categorisation and ultimately identifying or mitigating bias in datasets [21]. The implication of biased data on trained models is well documented, particularly in health [22].

A standardised assessment of an AI system’s performance, the readiness of clinicians to trust outputs, and the ethical acceptance by all concerned stakeholders are all essential requirements for the wider use of these systems, particularly in areas where the tools will help with clinical decision making such as for breast cancer detection. Recent standardisation efforts [23,24] are resulting in guidance for evaluating and monitoring AI systems. Projects such as ‘Developing a metrological framework for assessment of image-based artificial intelligence systems for disease detection’ (MAIBAI) [25] are developing impartial frameworks for performance, generalisability, and suitability assessment of AI tools, to ensure the safe use of AI software in a clinical setting. The evaluation of AI systems needs to be tailored to the specific task; for example, a screening system may need different evaluation than other classification tools in diagnostics due to the difference in the data (where we assume significantly more healthy cases in the general population). The associated importance of false positives and negatives also differ with the specific task, impacting the correct choice evaluation metrics and optimal operating point. The balance of referral rate and sufficiently high (in an ethical and financial sense) positive predictive value are all points of careful consideration in medical screening [26].

Accurate measurement and categorisation of data are critical for the quantitative validation and reliability assessment of AI models in healthcare imaging applications. In medical imaging, and mammography in particular, diverse clinical and technical features introduce significant heterogeneity that can affect the appearance and interpretation of images and ultimately confound the outputs of AI-based decision-support systems. This heterogeneity occurs between scanners, but also within the same scanner following different image processing routines or protocols or software updates. Standardised measurement frameworks for image metadata, acquisition protocols, patient population characteristics, and device-specific imaging parameters are essential to ensure traceable, equitable, and robust model development and evaluation. Recent advances in measurement science emphasise the necessity of systematic, reproducible quantification of image features, acquisition conditions, and population subgroups for AI evaluation in clinical imaging tasks [27].

Differences between patient subpopulations are observed through healthcare applications, including mammography. Several studies have investigated patient-level features such as association between demographics and breast cancer [28,29], or the impact on imaging of factors such as age and density [30]. Other studies have looked at the factors affecting image acquisition and quality [31,32,33], or how differences can impact AI models, such as mammography systems [34] and breast density [2]. However, consistent categorisation of data in studies is lacking and this has a direct impact on AI model training and evaluation, and ultimately the generalisability, reproducibility and fairness of AI models in healthcare.

This paper aims to develop and demonstrate a methodology for comprehensive measurement and categorisation of mammography imaging data, forming the basis for interpretable evaluation of AI systems in breast cancer screening. The approach assesses key measurement dimensions including compressed breast thickness, tissue composition, lesion morphology, technical acquisition settings (including image processing), and demographic variation, with the goal of providing a measurement-centric framework for dataset construction, bias detection, and performance benchmarking. By incorporating measurement science principles into the process of data subcategorisation, this work establishes quantitative protocols to improve the reliability and generalisability of AI models used in mammographic analysis.

Section 2 of this paper introduces a categorisation framework for mammography, distinguishing three groups: clinical, technical, and screening-process-related. It also describes the representative dataset used to evaluate each category. Section 3 reports the dataset statistics for these categories, while Section 4 discusses the main results. Finally, Section 5 provides concluding remarks.

## 2. Materials and Methods

### 2.1. Categorisation for Mammography

Due to the complex nature of disease progression, breadth of client histories and demographics (we note here that we refer to a patient throughout this article as a client—this is the terminology adopted by the database we use in our case study; subjects are not ill and thus are not patients and the noun ‘women’ is not deemed inclusive) [35], and an array of data acquisition system characteristics, it is critical when developing AI software to look at different subsets of the data for a given task, e.g., detecting a cancer in dense versus less dense breasts. Mammographic images are routinely stored as Digital Imaging and Communications in Medicine [36] (DICOM) files, an international standard for medical imaging information. These files contain metadata such as brand and type of mammography unit, imaging and client-related data including compressed breast thickness and settings (exposure factors, potentially including image processing settings, etc.) and software version. In some cases, the metadata includes the presence of breast implants, but this may be missing, and images can be linked to other data with the outcome of the screening exam. Many of the data elements in DICOM files can make easy identification of relevant data subsets possible. It is worth noting, however, that some DICOM fields are not always populated consistently (e.g., the presence of an implant), and others may be captured in vendor-specific ‘private’ tags as opposed to open standard tags; this should be taken into consideration when categorising data.

One of the challenges for AI software is the heterogeneity of the breast and differences in system and exposure factors used in mammographic X-rays leading to differences in appearance of mammograms. Any AI software will need to be adaptable for the full range of breast types, characteristics of all mammographic equipment and versions of acquisition and processing software to ensure generalised and equitable performance. It is necessary to check that software is not biased against certain subgroups. The following subsections describe the factors that are likely to affect the appearance of mammogram images and should therefore be considered in AI studies to ensure training data are not biased.

#### 2.1.1. Clinical Categories

##### Compressed Breast Thickness

In mammography, breasts are compressed to reduce the amount of radiation required to make an X-ray image and to enhance the visibility of lesions, which tend to have a different stiffness from healthy breast tissue. There is a wide range of compressed breast thickness (CBT) and area for women [37]. The CBT is defined as the height of the compression paddle above the breast support table when the breast is fully compressed during imaging. Worldwide, the majority of CBTs are between 20 mm and 100 mm [38]. The average CBT will be population-, mammography-view- and potentially equipment-dependent. The image quality and appearance will be affected by the CBT; a longer exposure is needed for larger CBTs, which may allow more breast movement during the exposure resulting in extra blurring, and will also require higher energies that will reduce the radiographic contrast between tissues, making cancers more difficult to spot [33]. Moreover, for larger CBTs, due to imaging limitations and the need to keep the radiation dose acceptable, a lower image quality is accepted. There are also differences in appearances as larger breasts will tend to be more fatty as opposed to thinner breasts that tend to be more glandular. The issues mentioned here potentially lead to differences in image perception, which might influence the output of AI algorithms. These dependencies mean that subgrouping the data in breast thickness classes is required when evaluating the performance of AI algorithms. 

##### Fibro-Glandularity

Breasts are made up of different kinds of tissues but are often characterised by the two main tissue types: adipose and fibro-glandular. There is a great deal of interest in the amount and distribution of these tissues in individual breasts as it is assumed that breast cancer originates from glandular tissue, making the amount of such tissue a risk factor for breast cancer [39]. Furthermore, its attenuation is very similar to that of certain types of tumours, meaning that in high-glandularity regions of the breast, tumours might be masked, potentially justifying the use of additional imaging techniques [40]. Broad classifications of fatty, scattered glandularity, heterogeneously dense and very dense tissue types are made to estimate the risk of breast cancer and the risk of masking of tumours. It is useful therefore to differentiate subgroups of women depending on breast density and morphology.

##### Types of Cancer: Masses, Calcification Clusters

**Figure 1:**
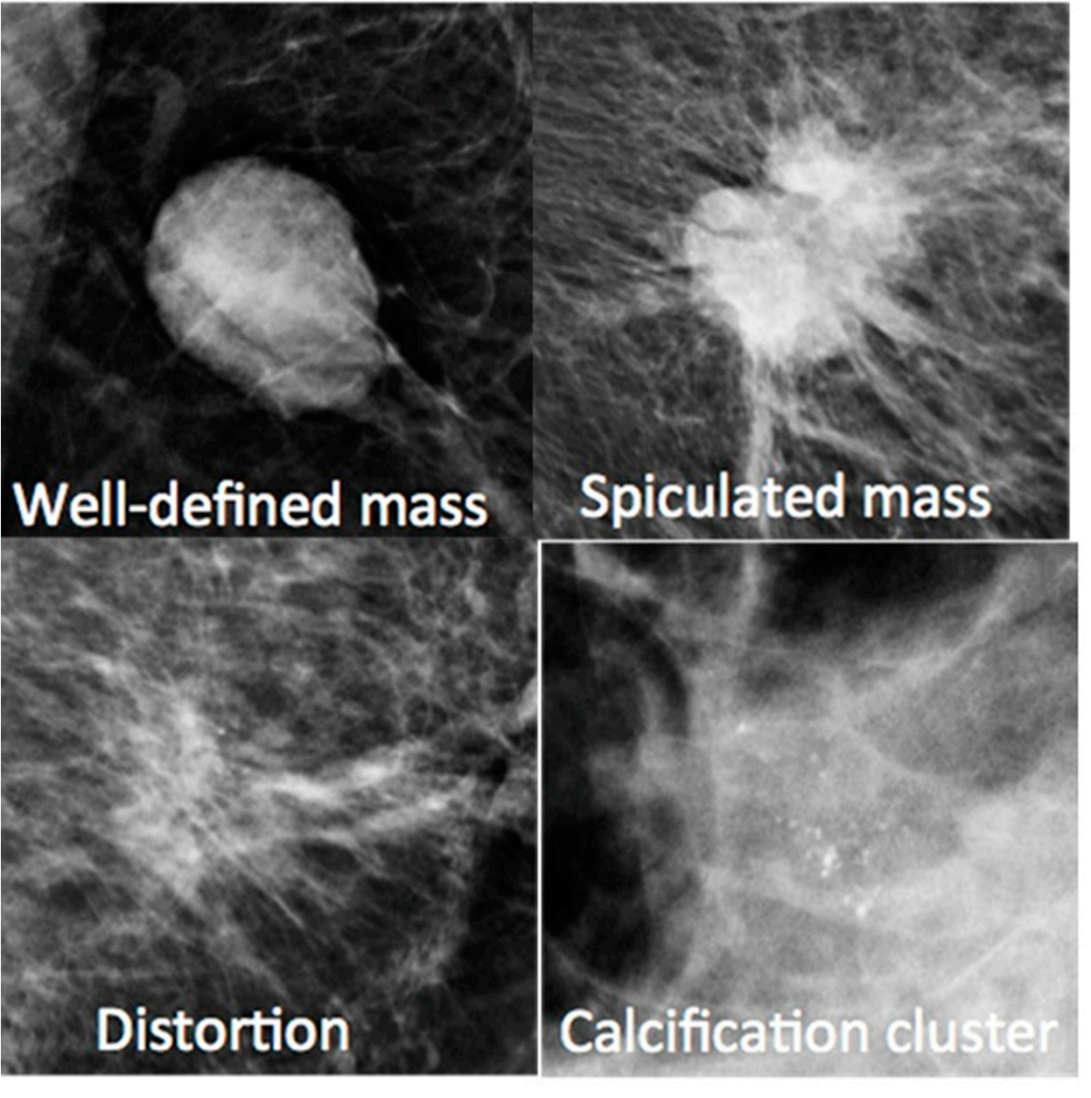
Examples of a well-defined and a spiculated mass, a distortion and a calcification cluster. Data from the OPTIMAM database [41].

Cancers will present themselves on mammographic images with different appearances as shown in Figure 1. Observation of the morphology of the mass therefore is important for the classification of the suspect structure—morphology may be classified as well-defined, spiculated or ill-defined. Sometimes, the cancer itself is not visible on the image but the presence of a lesion can be determined by local distortions in the images, caused by the difference in stiffness of the lesion and normal tissue. As the left and right breasts are part of the same organ, they are often broadly symmetrical. Differences between the left and right breast are therefore indicative of a lesion; this is called an asymmetry in the images of both breasts. As the attenuation of fibro-glandular tissue is very similar to that of cancerous masses, they will present as very similar pixel values (the same ‘whiteness’ on the mammography image) and therefore affect conspicuity.

Another indicator of tumours is the presence of calcification clusters [41]. These are groups of specks of calcium compounds and are not cancers in themselves but may be an indication of cancer, depending on the grouping and morphology of the calcifications. Therefore, training data including the subgrouping of different kinds of cancers and calcifications would be required for the evaluation of AI software and also to enable the computation of model confidence for asymmetric and calcification indicators. 

##### Types of Benign Features

Benign structures will present on mammographic images and sometimes resemble malignant structures. For example, one major difference in the appearance between a mass and a cyst is the edges of the structure, which are generally fuzzy or spiculated for a tumour and sharp for a benign structure. However, some image processing involves sharpening or blurring techniques, in which case the edges of a mass and cysts are more alike, or make areas resemble small calcifications. Image processing can therefore change the appearance of structures, potentially influencing the detection or characterisation of the structures as benign or malignant [42]. The threshold for identification decisions used to separate these features will impact the number of false positives and false negatives for the model, the cost of which differs for different clinical applications. A key element for the mammographic screening practice is to correctly ascertain a balance between disregarding minimal signs, potentially missing some early-stage cancers, and being risk averse, leading to an unnecessary high number of recalls. Different kinds of benign structures therefore should be a separate subcategory in the data and considered when evaluating AI software.

##### Population Differences

For reasons of equity, subgroups depending on ethnicity and age should be defined when evaluating AI software. Evidence is growing about the lack of generalisability of AI models across different subgroups and image presentation in healthcare, which in part is a result of the training data typically being biased or heavily weighted to a small number of subgroups [43]. This lack of coverage in the training data can have similar consequences to those seen in severe class imbalance classification problems, where a ‘good’ performance metric can be achieved by assigning all predictors to the majority class as opposed to learning the underlying features in any of the subgroups in the data. There are data which support differences in the occurrence of breast cancer [44] and there might be differences in breast types depending on ethnicity [45]. It is not exactly known what causes these differences, but this may be due to genetics, environmental factors, or other reasons. Therefore, appropriate coverage in the training and evaluation data is essential to obtain robust, generalisable, and equitable AI models.

The appearance of a mammogram will change as the client matures [46]. After the menopause, for example, breasts will tend to lose fibro-glandular tissue, hence appearing less dense. However, the use of hormone replacement therapy also changes the density of breasts, thus it could be suggested that women who are on hormone replacement therapy form a separate subgroup. Furthermore, it should be noted that radiographers routinely compare the past scans of a patient with their most recent, an aspect of work beginning to be incorporated in some models [47]. We illustrate population differences in our case study.

#### 2.1.2. Technical Features

The design features and characteristics of the mammography systems will affect the image appearance [48]. The energy and spectrum of the X-ray beam will be affected by the anode and filter used in the X-ray tube and the tube potential selected. These factors will affect the contrast within the image. The type of image detector will affect the sharpness, magnitude and appearance of noise, as will the use of an anti-scatter grid. Differences in the factors mentioned relating to system type could be subcategorised based on specification of the mammography system.

##### Equipment

There are several compression paddle types and sizes used in mammography, which can affect the final image [49]. The paddle size can be chosen according to the size of the breast, and will also affect the X-ray beam area, e.g., some of the support structure of the small paddles might be seen in the clinical image. There are also flexible paddles or paddles with curved shapes to match the cranial–caudal view of the breast. These will influence the distribution of the breast tissue; the thickness of the breast will be position-dependent and corrections are applied in the image processing, thus affecting its appearance in the image. Therefore, the subcategories of images made with different compression paddles should be identified when training and evaluating AI software.

The mammography systems are set up to ensure that there is sufficient dose to the detector to acquire clinical images with sufficient image quality. Generally, the higher the dose, the better the image quality. If the dose level is too low, then the images will be noisy and potentially obscure details. However, higher doses will generally also give a higher radiation dose to the woman, raising the health risks. Some suppliers offer the option to the user to select the dose level and overall setups of mammography equipment with respect to dose differs between countries [50]. These differences lead to differences in image quality for the same model of X-ray unit at different locations. 

##### Image Processing

An acquired image on a mammography unit is first corrected for defective detector elements, and a flat field correction and sometimes geometric distortions are applied. The resulting image after these corrections is often called the ‘for processing’ image. This image consists of a large dynamic range that is not useful for clinical diagnosis as the human eye can only appreciate a very limited number of contrast levels. Pixel values therefore need to be transformed into a reduced dynamic range and, at the same time, the structures in (local areas of) the breast need to be optimised for viewing. In this image processing, contrast is optimised and sharpening and/or noise reduction techniques may be applied. The output image is referred to as the ‘for presentation’ image. Each manufacturer has their own proprietary processing software and often more than one version of the software is in use in different places. In addition, the processing algorithms have multiple settings [51,52], leading to a different appearance of the image. These software versions and settings may also be periodically updated which may affect the perception of the image. Some suppliers adjust image processing to suit local preferences in image perception, which are subjective. This means that many image processing packages with different parameter settings may be changed over time, but it also means that historical images will have different processing from that in current use, introducing additional sources of variance into the data. As image processing has a major influence on the appearance and image content of the clinical images read by the clinical experts [33], subcategories with different processing (parameters) should be made when evaluating AI software. 

##### Breast Positioning

Image processing might be adversely influenced by suboptimal positioning, e.g., the presence of a large fold in the skin will lead to major artefacts in the image and a different perception of the image [53]. Clarity can also be compromised due to suboptimal positioning where part of the breast is not on the image. As this might influence the outcome of AI algorithms, a subcategory of images with suboptimal positioning should be included when evaluating AI software.

Typically, if there is a major artefact or an image is not of diagnostic quality, there will be additional imaging either on the day (technical repeat), or a woman would be recalled for further imaging at a later date (technical recall). Being able to identify and exclude images that are not of diagnostic quality may be important for an AI tool, though this also has wider applications than just breast positioning.

#### 2.1.3. Screening Process

##### Mammographic Views

National screening programmes may vary in the number of views acquired of the breast. In most European countries, and in the US, Canada, Australia and New Zealand [54], for example, the screening programmes have two standardised views: cranial–caudal (CC) and medio-lateral oblique (MLO). However, in other countries such as Japan, India and Pakistan before the year 2000 they would typically only take one view.

**Figure 2:**
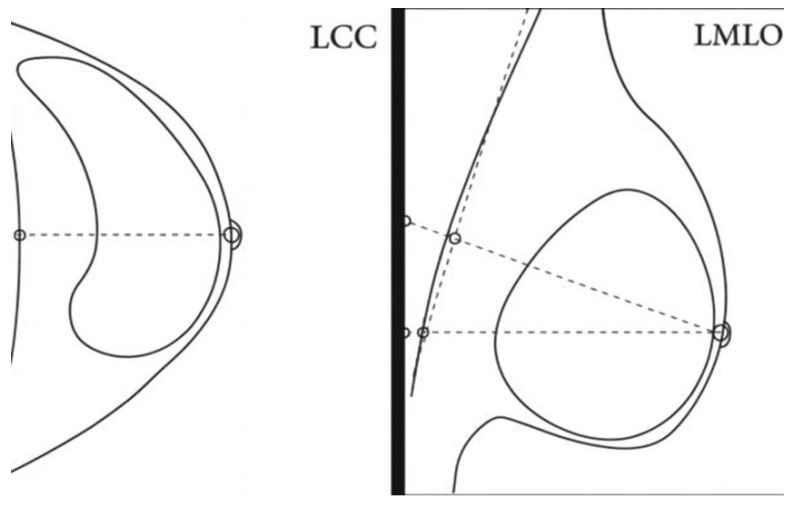
Schematic of CC and MLO views of a left breast (figure adapted from [51]).

During screening in the UK [55], typically four images are acquired for each woman: a CC view and an MLO view for each breast (see Figure 2). These two views are acquired partly to see cancers from two angles, but also because cancers are often better visualized in CC while the MLO images will show more of the tissue of the breast [56]. Furthermore, standard practice is to acquire two views to reduce the number of false positives due to the projection of normal overlying tissue in both views.

There are several reasons why women may not have four images present, including mastectomy or other surgery resulting in only one breast being present, or a refusal to undergo further imaging after imaging one breast due to the pain caused by breast compression. Also, women with very large breasts may need multiple images to cover one full breast, called mosaicking. Sometimes images will need to be repeated as there may have been an issue with one of the images. For some women different views than CC or MLO could be used, though this is typically more common in assessment imaging.

As the appearance of the breast is different in each view [57], AI software might respond to, and perform differently on, different perspectives. Therefore, subgrouping according to the number and categorisation of mammographic views seems a vital element in the evaluation of AI software. 

#### 2.1.4. Other Categories

##### Medical Devices

Clips, markers, or medical devices such as pacemakers are present in some mammograms [58]. Clips and markers are made of metals such as titanium, which are used to mark the location of suspicious regions in women that undergo a biopsy. They are used to help identify regions where a cancer may be or to show that an intervention has occurred. The clips or markers will be visible on the image alongside some scar tissue around it.

Depending on its type and location, a medical device may be seen on the mammogram. The presence of clips, markers and medical devices in the breast might influence the exposure during imaging and might cause image processing artefacts, both leading to a different appearance of the mammogram which potentially influences the result of AI algorithms.

##### Implants

Some women have breast implants (of silicone, a ‘bag’ filled with a saline solution, or made of different materials) [59], either for cosmetic reasons or following mastectomy. These breasts are more difficult to image because the implants tend to have a much higher attenuation than breast tissue. Therefore, the implant appears much whiter than the surrounding breast tissue which might obscure large parts of the image and might influence image processing. This is dependent on the placing of the implants (behind the pectoral muscle or before the pectoral muscle). The presence of implants also influences the compression of the breast as the compression tends to be lower in the presence of implants. Images with a breast implant will appear to be very different from standard mammography and are more difficult to read.

As the presence of implants has a major influence on the appearance of the mammographic images, making subgroups on the presence of implants is required especially in the training of AI models. We see this variation in our case study; see Section 3.4.

### 2.2. Available Mammography Image Datasets

There are a number of mammography datasets available to researchers and these range from fully open-source to requiring an author request. Table 1 shows client age and tumour type are frequently recorded among datasets; however, there appears to be more of a geometric and descriptive focus rather than patient-centric metadata. It is possible that such datasets are functional observations by and for clinicians, and less curated for research. Verification methods are also commonly recorded in this raw form as researchers would naturally filter what was deemed unreliable before modelling.

**Table 1:** Segmentation categories for mammogram datasets, including (i) Digital Database for Screening Mammography (DDSM) [60]; (ii) Curated breast imaging subset of DDSM [61]; (iii) Mammographic image analysis society (MIAS) [62] and mini-MIAS [63]; (iv) Breast Cancer Digital Repository (BCDR) [64]; (v) INbreast [65]; (vi) OPTIMAM [35]; (vii) Chinese mammography database (CMMD) [66]; (viii) VinDr-Mammo [67]; and (ix) CESM [68].

*Dataset*	*DDSM*	*Subset of DDSM*	*MIAS* *& Mini-MIAS*	*BCDR*	*INbreast*	*OMI-DB*	*CMMD*	*VinDr-Mammo*	*CESM*
Patient ID	X	X	X	X	X	X	X		X
Left/Right Breast		X				X	X	X	
Date of Study	X					X			
Age	X			X	X	X	X		
Ethnicity	X					X			
Density	X	X						X	
Mass Type	X	X	X		X		X		X
Verified	X	X		X	X	X	X		
# of Abnormalities	X	X				X	X		
Region of Interest	X	X	X	only + ve case	X	X	X		X
BI-RADS	X	X		X		X			X
Subtlety Rating	X	X				X			
Shape									X
Family History					X	X			
Clinical History				X	X	X			
Background Tissue	X	X	X			X			X
Size			X			X			
Subtype						*X*	*X*		

#### The OPTIMAM Mammography Image Database: A Case Study

For our case study we use the OPTIMAM Mammography Image Database (OMI-DB) which is a resource open to approved researchers containing mammography images from breast screening centres in the UK, complete with annotated cancer cases and clinical information [38]. Screening and prior mammograms—containing “for presentation” and in some cases “for processing” images—have been gathered for all screen-detected, interval cancers and large numbers of routine recall cases from multiple screening centres across the UK. All mammography images and data related to initial screening attendance, further assessments, and surgical outcomes are collected as part of a screening episode. Data collection for OMI-DB began in 2011 from two centres and data from more centres has been added since. As of 2025, OMI-DB contains data from thirteen centres [69]. A description of contents for all image-related information (DICOM header and expert annotations) as well as clinical observations for OMI-DB data are available here [70].

For this work, a dataset of 40,000 clients from six of the screening centres was created from OMI-DB, which was filtered to only include screening episodes from 2016–2022. The set was selected to have a large number of malignant cancers, and, for clients with multiple episodes in that timeframe, a single episode ID was selected at random to avoid duplication of client statistics. Here, we use only those images in the database labelled ‘for presentation’ and remove any images taken with smaller-than-standard paddles or MagView images.

DICOM header tags as well as information about the client were extracted using the Python pydicom package 3.0.2 [71] and the distribution of clients examined according to the categories identified in the previous sections. When selecting metadata from a database like OMI-DB there is a trade-off between including sufficient data in the training set to build the model versus having sufficient samples in the testing set to robustly evaluate the performance for minority classes.

## 3. Results

This section presents data characterisation statistics from the dataset described in Section 2.2. Where possible, categories identified in Section 2 have been analysed, to show how an AI system may be affected by a real-world example dataset. The impact on the AI system may be on the performance, generalisability and fairness of the model, all of which are contingent on the training data. We stress that we are using the previously described dataset from OMI-DB as a representative case study to highlight some of the potential implications in blindly using large datasets without consideration of the metadata (or data provenance) in the context of the specific task or research question. As a large database with well-curated metadata, we can contextualise the real-world implications of the areas listed in Section 2, applicable to not only mammography or health data, but to all AI models trained on recorded datasets. We also note that this work should be interpreted as acknowledging potential issues in training AI models, as opposed to critical reference to the OMI-DB data specifically.

### 3.1. Clinical Categories

Table 2 shows the outcome of clients from the dataset. Categories B and B+ refer to benign lesions, M and M+ are malignant lesions. For those with a malignant outcome (M and M+ in Table 2), Table 3 lists the highest grade of cancer found per client. Both Table 2 and Table 3 show a significant imbalance in the data distribution which is known to impact model training, and some performance metrics can be misleading in some cases. Highly imbalanced data requires carefully selected training, validation and testing data, as well as appropriate training schemes to prevent the majority class from dominating the model’s learning capacity. It is however also important to take into consideration the purpose of the tool. Applications such as screening programmes typically will exhibit a large imbalance in classes as most instances are likely to be ‘normal’, whereas in other applications, e.g., differentiating disease, classes may be more balanced.

**Table 2:** Distribution of client outcomes. B = benign; CIP = prior to interval cancer; M = malignant; N = normal; NA = normal with assessment; NAB = normal with assessment and biopsy; ‘+’ indicates annotations are available; cf. Section 2.1.1.

Client Outcome	Number of Clients	Percentage of Clients (%)
**B**	6970	17.5
**B+**	971	2.4
**CIP**	993	2.5
**M**	4781	12.0
**M+**	3197	8.0
**N**	21,360	53.6
**NA**	1548	3.9
**NAB**	67	<1
**Not recorded**	3	<1

**Table 3:** Distribution of lesion grades for clients with outcome M or M+. G1 to G3 are invasive cancer grades 1 to 3. NDH = high-risk ductal carcinoma in situ (DCIS) grade, NDI = intermediate-risk DCIS grade, NDL = low-risk DCIS grade (according to NBSS Crystal Report v5.4 [72]); cf. Section 2.1.1.

Malignant Lesion Grade	Number of Clients	Percentage of Clients
G1	984	12.3
G2	2312	29.0
G3	752	9.4
NDH	683	8.5
NDI	396	5.0
NDL	109	1.4
Not recorded	2721	34.1
Other	21	<1

**Figure 3:**
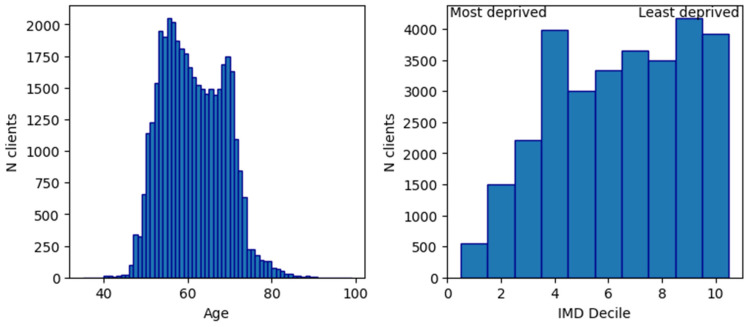
Age and deprivation data spreads. (**Left**) Distribution of client age in the dataset. Total clients = 39,874, missing clients = 16. (**Right**) Distribution of clients across index of multiple deprivation (IMD) deciles. Total clients = 29,795, missing clients = 10,095.

The UK breast cancer screening program focuses on women aged 50–70 [73] with some locations trialing a wider 47–73 age range [74]. Figure 3 shows the distribution of ages of clients in the dataset, with 86% of the data lying within the national screening focus range. The distribution appears to be bimodal with peaks at 55 and 69 and asymmetric tails due to the different reasons for the screening; for example, clients screened at younger ages are likely to have a family history of cancer. Women older than 70 are not invited to screening but they can self-refer. As cancer and other conditions can increase in prevalence with age, uniform random sampling of data may not be appropriate depending on the specific task.

Figure 3 also shows the distribution of clients by index of multiple deprivation (IMD) decile, where the 10th decile is the 10% of regions in the UK which are least deprived and the 1st decile is the 10% of areas in the UK which are most deprived, using multiple deprivation metrics. The distribution is heavily skewed towards least deprived areas, due to the concentration of clients in the dataset living in the Southeast of England where IMD deciles are higher. This means that people from more deprived areas are under-represented, potentially impacting the fairness of trained models. Moreover, this could have further implications in model fairness, as there is a correlation between deprivation and obesity [75], and obesity is correlated with breast size [76] and adverse cancer outcomes [29].

Table 4 shows the distribution of ethnicities in the dataset. The majority of clients are white, but a significant proportion of clients do not have a recorded ethnicity. Lack of data recording may be an issue for an AI model as it is important to train on representative samples of subgroups in the data being classified. As the physiologies of women can differ between ethnic groups (for instance, the distribution of breast size and density of (white) Western and Asian women are different), image data where ethnicity is not recorded may be excluded from, or be inappropriately used in, an AI training set. Ethnicities are self-declared so there is also some uncertainty in categorisation.

**Table 4:** Distribution of clients across ethnic categories.

Ethnic Category	Number	Percent
White	21,777	54.6
Not recorded	13,713	34.4
Asian/Asian British	1637	4.1
Black/Black British/Caribbean/African	978	2.5
Not stated	928	2.3
Any other ethnic group	530	1.3
Mixed/Multiple ethnic groups	327	<1

**Figure 4:**
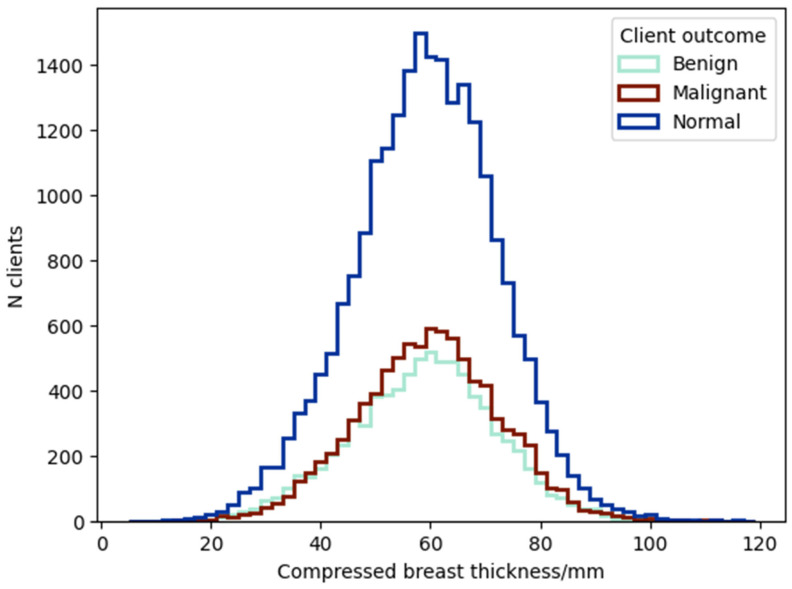
Distribution of compressed breast thickness by outcome. Missing: 0 clients; cf. Section 2.1.1.

The distribution of compressed breast thickness in clients is given in Figure 4. A Kolmogorov–Smirnov test indicates that the total distribution for all clients is not Gaussian (test statistic 0.0281, *p*-value << 0.0001), supported by low positive excess kurtosis (0.09) indicating a slight deviation from Gaussian. There is also a slight asymmetry in the distribution, which has a negative skewness (−0.07), indicating that the left tail (lower compressed breast thickness) is longer (i.e., larger) than the right tail (higher compressed breast thickness). Due to the non-Gaussian nature of the distributions, we estimate the mean and standard deviation, with the associated uncertainties of these estimates, using the bootstrap method with 200 bootstrap samples with replacement and confidence level 0.9. For all clients we obtain a mean of 58.57 ± 0.07 mm, differing slightly from the median of 59.00 mm due to the marginal asymmetry in the distribution, and standard deviation 13.05 ± 0.05 mm.

### 3.2. Technical Features

Table 5, Table 6 and Table 7 show the proportion of studies in the dataset that were imaged with different software versions for different manufacturers. Overall, there are 28,655 Hologic studies (71.3%), 8615 Siemens studies (21.4%) and 2934 GE studies (7.3%). Using different software, or indeed different versions of the same software or different image processing routines, may result in differences in the processing of mammography images and how they appear to clinicians or an AI tool. Different software versions may correlate with time due to systematic updates. It is important when creating a training set that software version, as well as any image processing settings, are considered as the differences in image appearance may affect the performance of the AI tool.

**Table 5:** Hologic mammography systems: software and version numbers; cf. Section 2.1.2 and Section 2.1.3.

Software Version	Number of Studies	Percentage of Total Studies
Lorad Selenia 3.4.1.8/1.4.0.7/1.7.3.10	18,431	45.8
Lorad Selenia 3.4.1.8/1.4.0.7/1.7.4.7	906	2.3
Lorad Selenia 3.4.1.8/1.4.1.0/1.7.3.10	1233	3.1
Lorad Selenia 3.4.1.8/2.0.0.2/1.7.3.10	958	2.4
Lorad Selenia 3.4.2.9/1.4.1.0/1.7.4.7	806	2.0
Selenia Dimensions 1.3	136	<1
Selenia Dimensions 1.7	712	1.8
Selenia Dimensions 1.8	1015	2.5
Selenia Dimensions 1.9	2248	5.6
Selenia Dimensions 1.10	2003	5.0
Selenia Dimensions 1.11	198	<1
Other	13	<1

**Table 6:** Siemens mammography systems: software and version numbers; cf. Section 2.1.2 and Section 2.1.3.

Software Version	Number of Studies	Percentage of Total Studies
VB41B	7434	18.5
VC20C	505	1.3
VB60D	296	<1
VB60C	183	<1
VA20F	168	<1
Other	29	<1

**Table 7:** GE mammography systems: software and version numbers; cf. Section 2.1.2.

Software Version	Number of Studies	Percentage of Total Studies
54.10	1440	3.6
53.40	841	2.1
55.30	521	1.3
55.41.3	99	<1
Other	38	<1

### 3.3. Screening Process

Multi-view mammography AI models, such as [43,77,78,79], expect four mammography images as input, namely, CC and MLO views of both the left and right breast. Of the 39,890 clients in the dataset, 36,862 clients had exactly four images, 2294 clients had more than four images, and 734 clients had fewer than four images. This means that 3028 clients (7.6%) could be excluded if using the multi-view AI models for mammography images. Of the 2294 clients with more than four images, 606 studies (1.5%) were technical recalls and 634 studies (1.6%) were technical repeats. In principle these clients could be used within a multi-view AI model; however, they would require manual selection of the four images for each study, and additional data processing steps for mosaic images of large breasts.

Table 8 shows the number and percentage of mammography views from studies in the dataset. Almost all studies in the dataset are the standard screening views, with the additional views relating to assessments, such as localisation of lesions. Although these are rare, well-curated data would enable these additional views to be excluded for evaluation with an AI screening tool as these would be out of distribution compared to the training data and hence the AI predictions may be unreliable.

**Table 8:** Mammographic views within the dataset. The majority of patients had the standard screening views MLO and CC, with a small fraction having supplementary views, such as the mediolateral (ML) or lateromedial (LM) view, or a CC image with implant displacement (CCID). Views with 10 or fewer studies have been grouped into “other”; cf. Section 2.1.3.

View Position	Number of Studies Containing This View	Percentage of Total Studies
MLO	40,096	99.7
CC	40,031	99.6
CCID	20	<1
LM	18	<1
ML	15	<1
Other	22	<1

### 3.4. Medical Devices and Implants

From 39,996 clients, with 808,585 images, 7134 images from 2605 clients (0.9% of images, 6.5% of clients) were found to have an implant or medical device that caused bright areas in the image. Of these 7134 images, 2916 images (40.9%) had the DICOM tag ‘BreastImplantPresent’ as True.

It is important to either remove images with implants or medical devices from any AI training set, or create an appropriate training subset for these images, as they may impact the results of the AI algorithm. The use of DICOM tags to flag the presence of an implant is not consistent which may make identifying implants in large datasets difficult and time consuming.

## 4. Discussion

The dataset analysed here is a subset of the OMI-DB. It is a relatively large data set using relatively recently acquired images. We showed it is a heterogeneous dataset, but it is possible to characterise the dataset and show where there are subgroups that may not have sufficient cases, e.g., extremes of compressed breast thickness, ethnicities and equipment manufacturers. The advantage of the OMI-DB is that some of these shortages can be improved by including more images, such as equipment. But this cannot be done for all shortages. It is vital that a dataset is characterised, either to indicate the relevance of the training set and/or the image set that the AI will be applied to. It is clear, even with a large dataset such as OMI-DB, there are some sub-groups that have small numbers. There are a number of other publicly available datasets [66,80,81] and reviews of the contents of some of these can be found in [82]. The largest of the datasets outside of OMI-DB is the Chinese Mammography Database (CMMD) [66] with 1775 cases, meaning that only broad sub-group analysis may be done. For these sub-groups appropriate strategies must be employed, such as strict inclusion/exclusion criteria, training schemes and tailored evaluation metrics. It should be noted that several of these datasets contain scanned films [60,62] which should be avoided for model training in general; these images are intrinsically different in look and quality from digital images.

The ethnicity of the publicly available datasets is primarily Northern European. The main exceptions are VinDr-Mammo [67] which has a Vietnamese population and CMMD [66] which has a Chinese population. The EMory BrEast Imaging Dataset (EMBED) [80] has a large African American population (42%). The physiologies of women can differ between ethnic groups, meaning AI tools trained on one dataset may not be easily applied to another. Ethnicity is important to consider when designing AI models; however, it can be difficult to categorise due to lack of recording, as shown in Section 3, leading to small subgroup sizes and insufficient data.

We demonstrated the distribution of compressed breast thicknesses in the dataset for three systems. Any differences that exist will be more likely due to the population rather than the imaging system. At the extremes of the range of breast thicknesses in Figure 4, there are some relatively small numbers. It will be difficult to train and evaluate the performance of AI with these numbers. At the extremes, the largest breasts will be more fatty but also many of them will require multiple images to cover the whole breast. The thinner breasts are more glandular and indeed image presentation for compressed breasts less than 20 mm thick have been shown to be poor [33]. Therefore, there is potential bias against women presenting with smaller or larger breasts than average. The average CBT presented here is 58.57 ± 0.07 mm. If this value is compared to CBTs for data collected between 2016 and 2019 for the whole of the UK, then we see that it is less than the national average of 60.7 mm [83] (average of reported CC and MLO). The population in the dataset is primarily from the South of England and perhaps indicates regional differences within the UK as there must be areas where the breasts are on average larger.

There is a bias in the data to women in less deprived areas. This may be partly due to the areas of data collection, but it may be difficult to avoid as it has been shown that there is an association between non-attendance at breast screening and deprivation [28]. The dataset is primarily for women between the ages of 50 and 70 years old, which matches the UK screening population. Many other national screening programmes will have different ranges of age. Age will affect the appearance of the mammograms; for example, the glandularity of women changes as they get older [30].

The age of the dataset is also important. There is an inevitability that AI will be trained on image sets that are older; firstly, time is required to collect sufficient images, and secondly, a true normal case will require a follow-up screen, in case the woman returns with an interval cancer and/or has an abnormal result following a screening event. The best datasets will still be a few years old at best but many will be considerably older [65]. It is interesting to note what differences time can make to the datasets. In addition to potential changes in practice or screening staff, it has been shown that the quality of imaging systems has improved over time [31]. Image processing changes have been made that affect the image presentation [52]. There are a variety of software versions contained within the database examined in this study. It should be noted that most of these changes will not affect the image presentation but may be changes to the interface or for reasons of security. Image processing settings are likely to produce larger differences in image presentation than software versions, as they can be designed to give images a different look [52]. We know that AI can be affected by the presentation of the image; for example, Vries et al. [34] showed recall rate increasing by a factor of four following a change in software version. Equipment type may also change over time; for example, the dataset CMMD [66] only contains GE Senographe DS, whereas these sets have been obsolete in the UK for over a decade.

Additionally, the population can change over time; it has been shown that the average compressed breast thickness has increased over time in the UK [83]. Datasets such as CMMD [66] were acquired prior to 2015. The OMI-DB database [41] is continually acquired and so the images can be quite recent in the database; however, due to the screening interval in the NHS, we do not know the ground truth of normal cases until three years passes and the woman returns for the next screen.

There can also be limitations on the cases that can be applied to screening images. AI products tend to be trained with four views [84] and so cases outside of this range may not be acceptable to the AI tool. Most of these are women who require multiple images to fully image the breast. There is a question of bias that there are sub-groups that may be poorly served, if at all, by AI. This is particularly of interest as we know that there are links between deprivation and obesity [75] and there is already health inequality in screening for deprived areas [28]. We have shown that 7.6% of clients in the database examined here may not be readable by AI due to not having the standard four views.

## 5. Conclusions

In this paper we have examined several factors affecting mammography imaging data. The specific categorisations discussed were (i) clinical—the types of masses identified; (ii) demographic—the differences between women; (iii) technical—the differences between imaging devices; and (iv) screening processes—the protocols for collecting images; along with other categories. This analysis reveals the complexity of breast screening images and associated metadata and provides a rationale for why AI models trained on datasets inappropriate for the task for which they are being developed may perform poorly and not generalise well. For AI to be fully trustworthy, it must be developed to be able to overcome these limitations. Work to this end has been carried out in the form of [85] and the New York University (NYU) model [48] where they use training data from different manufacturers.

We have described a wide range of factors that can be used to categorise an image set. There may be differences in the categories between image sets used for training of AI and in the field due to population and equipment setup. These differences may result in an AI product not working as optimally in the field as it did in its training and validation. While groups developing AI products may have their own private source of data they have collected [78,86], it is a difficult process to undertake securely and ethically, as shown by the methods required described in [87]. Therefore, it is easier to use other datasets that are publicly available. In this work, we have characterised the publicly available (under licence) OMI-DB dataset licenced to the MAIBAI project. We have shown that the dataset covers a wide range of sub-groups; however, there are still weaknesses in areas such as ethnicity (where the vast majority of women are white) and range of X-ray equipment (only three manufacturers with the majority being Hologic).

The importance of specific subcategories will vary depending on the task the AI is applied to, and the model. Some models may be more sensitive to some categories than others. We argue that it is important to test any model fully on available categories alongside a sensitivity analysis before deployment.

This concept will apply to other medical domains where AI models are trained on available, rather than appropriate, data for a given task. Work has already put into practice our findings by purposely selecting balanced datasets for training [27,88]. Further work will develop these ideas and directly measure differences in accuracy.

## Data Availability

The datasets presented in this article are not readily available because it is part of the OPTIMAM project, which has ethical approval to share its de-identified mammography images and clinical data with researchers, academics, and organizations, requires interested parties to submit an initial request via a web form that triggers a detailed application, followed by scientific review and critique by an OPTIMAM sub-committee, with all access granted subject to a CRT (Cancer Research Technology) sharing agreement since CRT owns the intellectual property for the de-identified image copies. See https://medphys.royalsurrey.nhs.uk/omidb/getting-access/ (accessed on 25 July 2026).

## References

[1] Kundu A.K., Doo F.X., Patil V., Varshney A., Jaja J. (2025). Detecting and Monitoring Bias for Subgroups in Breast Cancer Detection AI. arXiv.

[2] Larsen M., Olstad C.F., Lee C.I., Hovda T., Hoff S.R., Martiniussen M.A., Mikalsen K.Ø., Lund-Hanssen H., Solli H.S., Silberhorn M. (2024). Performance of an Artificial Intelligence System for Breast Cancer Detection on Screening Mammograms from BreastScreen Norway. Radiol. Artif. Intell..

[3] Albahri A., Duhaim A.M., Fadhel M.A., Alnoor A., Baqer N.S., Alzubaidi L., Albahri O., Alamoodi A., Bai J., Salhi A. (2023). A systematic review of trustworthy and explainable artificial intelligence in healthcare: Assessment of quality, bias risk, and data fusion. Inf. Fusion.

[4] Gichoya J.W., Thomas K., Celi L.A., Safdar N., Banerjee I., Banja J.D., Seyyed-Kalantari L., Trivedi H., Purkayastha S. (2023). AI pitfalls and what not to do: Mitigating bias in AI. Br. J. Radiol..

[5] Riley R.D., Ensor J., Snell K.I., Archer L., Whittle R., Dhiman P., Alderman J., Liu X., Kirton L., Manson-Whitton J. (2025). Importance of sample size on the quality and utility of AI-based prediction models for healthcare. Lancet Digit. Health.

[6] Ng A.Y. (2004). Feature selection, L1 vs. L2 regularization, and rotational invariance. Proceedings of the Twenty-First International Conference on Machine Learning (ICML ’04).

[7] Srivastava N., Hinton G., Krizhevsky A., Sutskever I., Salakhutdinov R. (2014). Dropout: A Simple Way to Prevent Neural Networks from Overfitting. J. Mach. Learn. Res..

[8] Miyato T., Maeda S.I., Koyama M., Nakae K., Ishii S. (2016). Distributional Smoothing with Virtual Adversarial Training. arXiv.

[9] Ntelemis F., Jin Y., Thomas S.A. (2024). A Generic Self-Supervised Framework of Learning Invariant Discriminative Features. IEEE Trans. Neural Netw. Learn. Syst..

[10] Xie Q., Dai Z., Hovy E., Luong T., Le Q. (2020). Unsupervised Data Augmentation for Consistency Training. Adv. Neural Inf. Process. Syst..

[11] Barnes J., Bartlett J.W., Wolk D.A., Van der Flier W.M., Frost C. (2018). Disease Course Varies According to Age and Symptom Length in Alzheimer’s Disease. J. Alzheimer’s Dis..

[12] Strand M., Bhatt S., Moll M., Baraghoshi D. (2024). Estimating effects of aging and disease progression in current and former smokers using longitudinal models. Glob. Epidemiol..

[13] Ageing and Health. https://www.who.int/news-room/fact-sheets/detail/ageing-and-health.

[14] Montégut L., López-Otín C., Kroemer G. (2024). Aging and cancer. Mol. Cancer.

[15] Van Herck Y., Feyaerts A., Alibhai S., Papamichael D., Decoster L., Lambrechts Y., Pinchuk M., Bechter O., Herrera-Caceres J., Bibeau F. (2021). Is cancer biology different in older patients?. Lancet Healthy Longev..

[16] Bianco A., Antonacci Y., Liguori M. (2023). Sex and Gender Differences in Neurodegenerative Diseases: Challenges for Therapeutic Opportunities. Int. J. Mol. Sci..

[17] Zalewska T., Pawelec P., Ziabska K., Ziemka-Nalecz M. (2023). Sexual Dimorphism in Neurodegenerative Diseases and in Brain Ischemia. Biomolecules.

[18] He Y., Su Y., Zeng J., Chong W., Hu X., Zhang Y., Peng X. (2022). Cancer-specific survival after diagnosis in men versus women: A pan-cancer analysis. MedComm.

[19] Wilkinson M.D., Dumontier M., Aalbersberg I.J., Appleton G., Axton M., Baak A., Blomberg N., Boiten J.W., da Silva Santos L.B., Bourne P.E. (2016). The FAIR Guiding Principles for scientific data management and stewardship: Comment. Sci. Data.

[20] Smith N.A.S., Sinden D., Thomas S.A., Romanchikova M., Talbott J.E., Adeogun M. (2020). Building confidence in digital health through metrology. Br. J. Radiol..

[21] Brochu-Williams F., Chrubasik M., Thomas S.A. (2023). A tool for curating and searching databases proving traceable analysis of data and workflows. Acta IMEKO.

[22] Alderman J.E., Palmer J., Laws E., McCradden M.D., Ordish J., Ghassemi M., Pfohl S.R., Rostamzadeh N., Cole-Lewis H., Glocker B. (2025). Tackling algorithmic bias and promoting transparency in health datasets: The STANDING Together consensus recommendations. Lancet Digit. Health.

[23] Smuha N.A. (2025). Regulation 2024/1689 of the Eur. Parl. & Council of June 13, 2024 (EU Artificial Intelligence Act). Int. Leg. Mater..

[24] bmirza_drupal_sso. FDA Issues Draft Guidance on Marketing Submissions for AI-Enabled Medical Devices|AHA News. https://www.aha.org/news/headline/2025-01-07-fda-issues-draft-guidance-marketing-submissions-ai-enabled-medical-devices.

[25] MAIBAI. https://www.maibaiproject.eu/.

[26] Bond M., Pavey T., Welch K., Cooper C., Garside R., Dean S., Hyde C. (2013). Systematic review of the psychological consequences of false-positive screening mammograms. Health Technol. Assess..

[27] Gao J., Wang Z., Zhao X., Yao X., Wei X. (2024). Surviving in Diverse Biases: Unbiased Dataset Acquisition in Online Data Market for Fair Model Training. Proc. AAAI/ACM Conf. AI Ethics Soc..

[28] Smith D., Thomson K., Bambra C., Todd A. (2019). The breast cancer paradox: A systematic review of the association between area-level deprivation and breast cancer screening uptake in Europe. Cancer Epidemiol..

[29] Carmichael A. (2006). Review article: Obesity as a risk factor for development and poor prognosis of breast cancer. BJOG Int. J. Obstet. Gynaecol..

[30] Checka C.M., Chun J.E., Schnabel F.R., Lee J., Toth H. (2012). The Relationship of Mammographic Density and Age: Implications for Breast Cancer Screening. Am. J. Roentgenol..

[31] Mackenzie A., Khan R., Young K.C. (2020). Historical trends in image quality and mean glandular dose in digital mammography. 15th International Workshop on Breast Imaging (IWBI2020).

[32] Sweeney R.-J.I., Lewis S.J., Hogg P., McEntee M.F. (2018). A review of mammographic positioning image quality criteria for the craniocaudal projection. Br. J. Radiol..

[33] Loveland J., Mackenzie A. (2024). Image quality in mammograms with low compressed breast thicknesses. 17th International Workshop on Breast Imaging (IWBI 2024).

[34] de Vries C.F., Colosimo S.J., Staff R.T., Dymiter J.A., Yearsley J., Dinneen D., Boyle M., Harrison D.J., Anderson L.A., Lip G. (2023). Impact of Different Mammography Systems on Artificial Intelligence Performance in Breast Cancer Screening. Radiol. Artif. Intell..

[35] OPTIMAM Mammography Image Database (OMI-DB)|Cancer Research Horizons. https://www.cancerresearchhorizons.com/licensing-opportunities/optimam-mammography-image-database-omi-db.

[36] DICOM. DICOM. https://www.dicomstandard.org.

[37] Khan-Perez J., Mercer C., Bydder M., Sergeant J., Morris J., Maxwell A., Rylance C., Astley S. (2013). PB.10: Breast compression, compressed breast thickness and volumetric breast density. Breast Cancer Res..

[38] Fife I. (1991). The physical dimensions of the compressed breast. Br. J. Radiol..

[39] Fibroglandular Density: What Are Dense Breasts?. https://www.breastcancer.org/risk/risk-factors/dense-breasts.

[40] O’GRady S., Morgan M. (2018). Microcalcifications in breast cancer: From pathophysiology to diagnosis and prognosis. Biochim. Biophys. Acta (BBA)-Rev. Cancer.

[41] Halling-Brown M.D., Warren L.M., Ward D., Lewis E., Mackenzie A., Wallis M.G., Wilkinson L.S., Given-Wilson R.M., McAvinchey R., Young K.C. (2021). OPTIMAM Mammography Image Database: A Large-Scale Resource of Mammography Images and Clinical Data. Radiol. Artif. Intell..

[42] Warren L.M., Halling-Brown M., Looney P., Dance D., Wallis M., Given-Wilson R., Wilkinson L., McAvinchey R., Young K. (2017). Image processing can cause some malignant soft-tissue lesions to be missed in digital mammography images. Clin. Radiol..

[43] Schopf C.M., Ramwala O.A., Lowry K.P., Hofvind S., Marinovich M.L., Houssami N., Elmore J.G., Dontchos B.N., Lee J.M., Lee C.I. (2024). Artificial Intelligence-Driven Mammography-Based Future Breast Cancer Risk Prediction: A Systematic Review. J. Am. Coll. Radiol..

[44] SEER*Explorer Application. https://seer.cancer.gov/statistics-network/explorer/application.html?site=1&data_type=1&graph_type=2&compareBy=sex&chk_sex_3=3&chk_sex_2=2&rate_type=2&race=1&age_range=1&hdn_stage=101&advopt_precision=1&advopt_show_ci=on&hdn_view=0&advopt_show_apc=on&advopt_display=2#resultsRegion0.

[45] Sechopoulos I., Dance D.R., Boone J.M., Bosmans H.T., Caballo M., Diaz O., van Engen R., Fedon C., Glick S.J., Hernandez A.M. (2024). Joint AAPM Task Group 282/EFOMP Working Group Report: Breast dosimetry for standard and contrast-enhanced mammography and breast tomosynthesis. Med. Phys..

[46] Ginsburg O.M., Martin L.J., Boyd N.F. (2008). Mammographic density, lobular involution, and risk of breast cancer. Br. J. Cancer.

[47] Park J., Phang J., Shen Y., Wu N., Kim S., Moy L., Cho K., Geras K.J. (2019). Screening Mammogram Classification with Prior Exams. arXiv.

[48] Schurz H., Solander K., Åström D., Cossío F., Choi T., Dustler M., Lundström C., Gustafsson H., Zackrisson S., Strand F. (2025). Simulating mismatch between calibration and target population in AI for mammography the retrospective VAIB study. npj Digit. Med..

[49] Moshina N., Sebuødegård S., Evensen K.T., Hantho C., Iden K.A., Hofvind S. (2019). Breast compression and experienced pain during mammography by use of three different compression paddles. Eur. J. Radiol..

[50] Naik S., Varghese A.P., Asrar Ul Haq Andrabi S., Tivaskar S., Luharia A., Mishra G.V. (2024). Addressing Global Gaps in Mammography Screening for Improved Breast Cancer Detection: A Review of the Literature. Cureus.

[51] Mackenzie A., Loveland J., Farshadi L., Roozemond C., van Engen R.E. (2026). A National Audit of Mammography Systems Settings That May Affect the Output of Artificial Intelligence Software. Diagnostics.

[52] Barufaldi B., Zuckerman S.P., Medeiros R.B., Maidment A.D., Schiabel H. (2020). Characterization of the imaging settings in screening mammography using a tracking and reporting system: A multi-center and multi-vendor analysis. Phys. Medica.

[53] Popli M.B., Teotia R., Narang M., Krishna H. (2014). Breast Positioning during Mammography: Mistakes to be Avoided. Breast Cancer.

[54] Mesurolle B., El Khoury M., Travade A., Bagard C., Pétrou A., Monghal C. (2021). Is there any added value to substitute the 2D digital MLO projection for a MLO tomosynthesis projection and its synthetic view when a 2D standard digital mammography is used in a one-stop-shop immediate reading mammography screening?. Eur. Radiol..

[55] Example of a Standard Examination Protocol. GOV.UK. https://www.gov.uk/government/publications/breast-screening-guidance-on-implementation-of-ionising-radiation-medical-exposure-regulations-2017/example-of-a-standard-examination-protocol.

[56] Positioning and Technique—Radiology|UCLA Health. https://www.uclahealth.org/departments/radiology/education/breast-imaging-teaching-resources/screening-mammogram/positioning-and-technique.

[57] Young K.C., Wallis M.G., Blanks R.G., Moss S.M. (1997). Influence of number of views and mammographic film density on the detection of invasive cancers: Results from the NHS Breast Screening Programme. Br. J. Radiol..

[58] Thomassin-Naggara I., Lalonde L., David J., Darai E., Uzan S., Trop I. (2012). A plea for the biopsy marker: How, why and why not clipping after breast biopsy?. Breast Cancer Res. Treat..

[59] Cosmetic Breast Augmentation: A Review of Mammographic Findings—ProQuest. https://www.proquest.com/openview/b81db1bca5939b532d6b14bd0244ec96/1?pq-origsite=gscholar&cbl=32662.

[60] Rose C., Turi D., Williams A., Wolstencroft K., Taylor C., Astley S.M., Brady M., Rose C., Zwiggelaar R. (2006). Web Services for the DDSM and Digital Mammography Research. Digital Mammography.

[61] Sawyer-Lee R., Gimenez F., Hoogi A., Rubin D. (2016). Curated Breast Imaging Subset of Digital Database for Screening Mammography (CBIS-DDSM), The Cancer Imaaging Archive. https://paperswithcode.com/dataset/cbis-ddsm.

[62] Suckling J., Parker J., Dance D., Astley S., Hutt I., Boggis C., Ricketts I., Stamatakis E., Cerneaz N., Kok S. (2015). Mammographic Image Analysis Society (MIAS) Database v1.21. https://www.repository.cam.ac.uk/handle/1810/250394.

[63] The Mini-MIAS Database of Mammograms. http://peipa.essex.ac.uk/info/mias.html.

[64] Lopez M.G., Posada N., Moura D.C., Pollán R.R., Valiente J.M.F., Ortega C.S., Solar M., Diaz-Herrero G., Ramos I.M.A.P., Loureiro J. (2012). BCDR: A Breast Cancer Digital Repository. 15th International Conference on Experimental Mechanics.

[65] Moreira I.C., Amaral I., Domingues I., Cardoso A., Cardoso M.J., Cardoso J.S. (2012). INbreast: Toward a full-field digital mammographic database. Acad. Radiol..

[66] CMMD. The Cancer Imaging Archive (TCIA). https://www.cancerimagingarchive.net/collection/cmmd/.

[67] Nguyen H.T., Nguyen H.Q., Pham H.H., Lam K., Le L.T., Dao M., Vu V. (2023). VinDr-Mammo: A large-scale benchmark dataset for computer-aided diagnosis in full-field digital mammography. Sci. Data.

[68] CDD-CESM The Cancer Imaging Archive (TCIA). https://www.cancerimagingarchive.net/collection/cdd-cesm/.

[69] Participating Centres—OMI-DB. https://medphys.royalsurrey.nhs.uk/omidb/about-omi-db/participating-centres/.

[70] Description of Contents—OMI-DB. https://medphys.royalsurrey.nhs.uk/omidb/description-of-contents/.

[71] (2026). *pydicom/pydicom*. Python. DICOM in Python. https://github.com/pydicom/pydicom.

[72] Breast Screening: Screening Office Management. GOV.UK. https://www.gov.uk/government/publications/breast-screening-screening-office-management/breast-screening-screening-office-management.

[73] Who Breast Screening Is for. nhs.uk. https://www.nhs.uk/tests-and-treatments/breast-screening-mammogram/who-breast-screening-is-for/.

[74] Evaluating the Age Extension of the NHS Breast Screening Programme. Health Research Authority. https://www.hra.nhs.uk/planning-and-improving-research/application-summaries/research-summaries/evaluating-the-age-extension-of-the-nhs-breast-screening-programme/.

[75] Stafford M., Brunner E.J., Head J., Ross N.A. (2010). Deprivation and the Development of Obesity: A Multilevel, Longitudinal Study in England. Am. J. Prev. Med..

[76] Steele J.R., Coltman C.E., McGhee D.E. (2020). Effects of obesity on breast size, thoracic spine structure and function, upper torso musculoskeletal pain and physical activity in women. J. Sport Health Sci..

[77] Zafari Y., Elalfy R., Mabrok M., Al-Maadeed S., Khattab T., Rashed E.A. (2025). A Hybrid CNN-VSSM model for Multi-View, Multi-Task Mammography Analysis: Robust Diagnosis with Attention-Based Fusion. arXiv.

[78] NYU Breast Cancer Dataset|PDF|Mammography|Biopsy. Scribd. https://www.scribd.com/document/755011860/NYU-Breast-Cancer-Dataset.

[79] Gastounioti A., Eriksson M., Cohen E.A., Mankowski W., Pantalone L., Ehsan S., McCarthy A.M., Kontos D., Hall P., Conant E.F. (2022). External Validation of a Mammography-Derived AI-Based Risk Model in a U.S. Breast Cancer Screening Cohort of White and Black Women. Cancers.

[80] Jeong J.J., Vey B.L., Bhimireddy A., Kim T., Santos T., Correa R., Dutt R., Mosunjac M., Oprea-Ilies G., Smith G. (2023). The EMory BrEast imaging Dataset (EMBED): A Racially Diverse, Granular Dataset of 3.4 Million Screening and Diagnostic Mammographic Images. Radiol. Artif. Intell..

[81] Aqdar K.B., Mustafa R.K., Abdulqadir Z.H., Abdalla P.A., Qadir A.M., Shali A.A., Aziz N.M. (2024). Mammogram mastery: A robust dataset for breast cancer detection and medical education. Data Brief.

[82] Logan J., Kennedy P.J., Catchpoole D. (2023). A review of the machine learning datasets in mammography, their adherence to the FAIR principles and the outlook for the future. Sci. Data.

[83] Loveland J., Mackenzie A. (2024). Radiation doses received in the UK breast screening programmes 2019–2023. Br. J. Radiol..

[84] Ibragimov A., Senotrusova S., Litvinov A., Ushakov E., Karpulevich E., Markin Y. (2024). MamT^4^: Multi-View Attention Networks for Mammography Cancer Classification. 2024 IEEE 48th Annual Computers, Software, and Applications Conference (COMPSAC).

[85] Wu N., Phang J., Park J., Shen Y., Huang Z., Zorin M., Jastrzebski S., Fevry T., Katsnelson J., Kim E. (2020). Deep Neural Networks Improve Radiologists’ Performance in Breast Cancer Screening. IEEE Trans. Med. Imaging.

[86] Dahlblom V., Dustler M., Bolejko A., Bakic P.R., Granberg H., Johnson K., Förnvik D., Lång K., Tingberg A., Zackrisson S. (2023). Malmö Breast ImaginG database: Objectives and development. J. Med. Imaging.

[87] Sourlos N., Vliegenthart R., Santinha J., Klontzas M.E., Cuocolo R., Huisman M., van Ooijen P. (2024). Recommendations for the creation of benchmark datasets for reproducible artificial intelligence in radiology. Insights Imaging.

[88] Hsu W., Hippe D.S., Nakhaei N., Wang P.-C., Zhu B., Siu N., Ahsen M.E., Lotter W., Sorensen A.G., Naeim A. (2022). External Validation of an Ensemble Model for Automated Mammography Interpretation by Artificial Intelligence. JAMA Netw. Open.

